# Multiple disconnection syndrome, interoceptive metacognition deficits and fatigue in multiple sclerosis

**DOI:** 10.1093/braincomms/fcae302

**Published:** 2024-09-04

**Authors:** Moussa A Chalah, Samar S Ayache

**Affiliations:** Institut de Neuromodulation, Pôle Hospitalo-Universitaire Psychiatrie Paris 15, GHU Paris Psychiatrie et Neurosciences, Hôpital Sainte-Anne, 75014 Paris, France; Department of Neurology, Gilbert and Rose-Marie Chagoury School of Medicine, Lebanese American University, 4504 Byblos, Lebanon; Institut de la Colonne Vertébrale et des Neurosciences (ICVNS), Centre Médico Chirurgical Bizet, 75116 Paris, France; Department of Neurology, Gilbert and Rose-Marie Chagoury School of Medicine, Lebanese American University, 4504 Byblos, Lebanon; Institut de la Colonne Vertébrale et des Neurosciences (ICVNS), Centre Médico Chirurgical Bizet, 75116 Paris, France; EA 4391, Excitabilité Nerveuse et Thérapeutique, Faculté de Santé, Université Paris Est, 94010 Créteil, France; Service de Physiologie-Explorations Fonctionnelles, DMU FIxIT, Hôpital Henri Mondor, 94010 Créteil, France

## Abstract

This scientific commentary refers to ‘Understanding the mechanisms of fatigue in multiple sclerosis: linking interoception, metacognition and white matter dysconnectivity’, by Danciut *et al*. (https://doi.org/10.1093/braincomms/fcae292).

This scientific commentary refers to ‘Understanding the mechanisms of fatigue in multiple sclerosis: linking interoception, metacognition and white matter dysconnectivity’, by Danciut *et al*. (https://doi.org/10.1093/braincomms/fcae292).

Fatigue is a frequent complaint in multiple sclerosis, affecting up to 90% of patients.^1^ It can occur from the early stages of the disease even prior to establishing the diagnosis and can have a drastic impact on patients’ functioning (social, professional and familial) and their quality of life.^1^ It seems to be very prevalent and could potentially generate an important clinical and economic burden, yet it is still relatively overlooked compared with other multiple sclerosis symptoms, in what comes of its definition, aetiology, evaluation and management.^1^ What is known is that multiple sclerosis fatigue is pathological, and it is different from tiredness or exhaustion reported by healthy individuals; several attempts have been made to provide clear and adequate definitions.^1^ Until now, researchers have pinpointed its multidimensional aspect with physical, motor and psychosocial components.^1^ In terms of evaluation, the available works have approached it either as a subjective perception using patient-rated scales (e.g. modified fatigue impact scale) or as an objective measure reflected by a time-dependent decline during the performance of a cognitive or motor task; the latter is usually referred to as fatigability rather than fatigue.^1^ From a mechanistic point of view, fatigue could stem from several factors such as anaemia, endocrinopathies, vitamin deficiencies, sleep disturbance, affective comorbidities, motor weakness or medication side effects, among others.^1^ This characterizes ‘secondary fatigue’. However, it could arise from a cerebral pathophysiological process related to multiple sclerosis (e.g. neuroinflammation, neurochemical imbalance and structural and functional cerebral abnormalities), and this denotes ‘primary fatigue’.^1^ In this context, it is worth noting that the neuroimaging studies have yielded heterogeneous results hinting towards structural and functional abnormalities in several grey and white matter regions. Despite the dearth and inconsistency of these data, a cortico-striato-thalamo-cortical loop of multiple sclerosis fatigue has been suggested.^1^ As for its management, it is still a challenging task with modest outcomes obtained with several clinical trials including pharmacological and alternative interventions.^1^ Such limitations in the field of multiple sclerosis fatigue warrant more research on this matter employing novel approaches, in order to further understand the complexity of this symptom and subsequently develop mechanism-based interventions.

Recently, a growing interest has been noted regarding the potential contribution of interoceptive and (meta)cognitive deficits to fatigue, particularly cognitive fatigue, with only very few works tackling this topic in the context of multiple sclerosis.^2,3^ Interoception, a term introduced by Sherrington in 1906, entails the perception of internal body signals.^4^ As for metacognition, defined by Flavell in the 1970s, it signifies the self-assessment of one’s own cognitive processes (i.e. cognition about cognition).^5^ Considering interoception and according to the allostatic self-efficacy theory, fatigue might result from chronic dyshomeostasis. In addition, general metacognitive deficits might arise from neuroinflammation and tissue damage, leading to altered metacognitive networks, which might also explain fatigue. Such a novel cognitive approach might help overcome the previous limitations and open a new venue for understanding and treating this debilitating symptom.

In this issue of *Brain Communications*, Danciut *et al*. elegantly investigated this relationship in neuroimaging and behavioural research involving 71 adult patients, predominantly females, with relapsing-remitting multiple sclerosis.^6^ In this observational and cross-sectional study from the UK, patients underwent specific MRI sequences, namely diffusion tensor, quantitative magnetization transfer and neurite orientation dispersion and density imaging, which permit depicting microstructural abnormalities. They also performed tasks assessing interoception (i.e. insight/awareness and accuracy using heartbeat tracking and discriminative tasks, respectively) and metacognition (i.e. visual perception and memory tasks). Measures of fatigue, quality of life and potential confounders were gathered including physical disability, affective symptoms, daytime sleepiness, cognition and treatment profiles.

Patients with high cognitive fatigue have a significantly lower quality of life compared with their counterparts with low cognitive fatigue, highlighting the prominent influence of this symptom on the patients’ perception of satisfaction. In fact, the altered quality of life in this population could be reflected by the frequency of unemployment or underemployment, divorce rate, reduced social participation and social isolation, among others. After running a binomial logistic regression controlling for clinical and socio-demographic variables (age, sex, disease duration, disability and affective symptoms), Danciut *et al*. observed higher odds of cognitive fatigue among patients with low interoceptive insights. The remaining outcomes (i.e. interoceptive accuracy and metacognitive measures) did not significantly predict high odds of cognitive fatigue. Therefore, cognitive fatigue in multiple sclerosis seems to be associated with a specific deficit in interoceptive insight—also known as the metacognitive aspect of interoception—rather than a general deficit in metacognition. Consistently, in a previous work by Rouault *et al*.,^2^ cognitive fatigue was significantly associated with interoceptive awareness but not with metacognition. Conversely, Gonzalez Campo *et al*.^3^ found a significant correlation between fatigue and interoceptive accuracy, but this could be attributed to the consideration of total fatigue score, which encompasses items assessing motor and psychosocial fatigue in addition to the cognitive dimension.

Besides regression analysis, correlation analysis between cognitive fatigue and MRI measures was also run by Danciut *et al*. This has unveiled significant inverse correlations between cognitive fatigue and each of fractional anisotropy (a marker of axonal damage) and neural density within several bilateral white matter tracts. This relationship remained significant in the case of neural density of several tracts after adjusting for relevant confounders (disability, depression and disease duration). Along with the absence of significant correlations with magnetization transfer measures that reflect inflammation and demyelination, this finding highlights the principal contribution of widespread axonal pathologies in the manifestation of cognitive multiple sclerosis fatigue. Interestingly, the involved white matter tracts seem to take part in interoception networks. Here, it is worth stating that multiple sclerosis could be regarded as a disease with multiple disconnection syndrome that could arise from the disruption of white matter tracts linking specific cerebral hubs and might lead to structural and functional changes within some networks.^7^ Once the damage exceeds what is considered the threshold of network efficiency, compensatory mechanisms can no longer take place, and a network collapse would occur.^7^ This could be incriminated in the expression of several symptoms including fatigue, motor disability, cognitive deficits and affective manifestations.^7^

Finally, when studying the effect of the interaction between cognitive fatigue and the behavioural measures on MRI parameters, Danciut *et al*. reported a significant interaction between cognitive fatigue and interoceptive insights, which further confirms that low interceptive insight drives the association between axonal pathologies in interoceptive insight network and expression of cognitive fatigue. In other terms, patients with multiple sclerosis presenting an extensive axonal involvement will exhibit lower interoceptive metacognition and will report high cognitive fatigue. This finding could have pertinent clinical implications. By acknowledging the contribution of interoceptive metacognition deficits in multiple sclerosis fatigue, efforts could be put into developing interoceptive metacognition-based interventions aiming to alleviate fatigued patients with multiple sclerosis.

In conclusion, the study by Danciut *et al*. highlights a dyshomeostatic theory of fatigue in multiple sclerosis. This work is interesting on two levels: firstly, it provides new insights into neural and behavioural underpinnings of cognitive fatigue in multiple sclerosis; secondly, it may help develop specific interventions targeting a difficult-to-treat complaint in multiple sclerosis.

Regarding the first (mechanistic) point, the relationship between multiple sclerosis fatigue, interoception and metacognition merits further consideration in future studies that could replicate the present finding and apply similar as well as complementary neuroimaging, neurophysiological and laboratory workup (e.g. functional MRI, volumetric analysis, spectroscopy, transcranial magnetic stimulation, high-definition electroencephalography and immune panel). Moreover, performing the study in patients with other disease types (progressive multiple sclerosis forms) where neurodegeneration predominates the pathophysiological picture could help unravel the contribution of this mechanism to the studied relationship. Furthermore, some multiple sclerosis symptoms tend to cluster together with fatigue, such as anxiety, depression and alexithymia (i.e. difficulties in differentiating one’s own emotions from bodily signals, difficulties in identifying and describing one's own emotions).^8^ Interoceptive deficits have been previously associated with these manifestations in psychiatric and healthy populations,^9,10^ a finding that deserves to be tested in patients with multiple sclerosis.

Regarding the second (therapeutic) point, based on Danciut et *al*.’ observations, it would be interesting to test the effects of some interventions targeting interoception on multiple sclerosis fatigue. The interventions could be applied as a monotherapy or in a combination fashion aiming to obtain a synergistic or a cumulative effect, and they could include but are not limited to pharmacotherapeutics, interoceptive technologies, mindfulness-based interventions, neurofeedback programs, interoceptive metacognitive training and non-invasive brain stimulation.^8,9,11^ Here, considering the involvement of white matter pathologies observed in the current work, it would be of interest to consider the emergent concept of white matter plasticity in addition to the well-known mechanism of synaptic plasticity.^12^ This emphasizes the role of early interventions in mobilizing the remaining compensatory mechanisms, supporting functional rerouting within brain networks and thus preserving certain functions or preventing the appearance of worrisome cognitive, affective and behavioural symptoms.

## Data Availability

No new data were created or analysed.
